# Unmasking epithelial-mesenchymal transition in a breast cancer primary culture: a study report

**DOI:** 10.1186/1756-0500-5-343

**Published:** 2012-07-03

**Authors:** Luigi Minafra, Rossana Norata, Valentina Bravatà, Massimo Viola, Carmelo Lupo, Cecilia Gelfi, Cristina Messa

**Affiliations:** 1 Institute of Molecular Bioimaging and Physiology (IBFM), National Council of Researches (CNR), Cefalù-Segrate, Italy; 2 Laboratorio di Tecnologie Oncologiche (LATO) – HSR G.Giglio, Cefalù, Italy; 3 Department of Surgery, San Raffaele G. Giglio Hospital, Cefalù, Italy; 4 Division of Pathology, La Maddalena Hospital, Palermo, Italy; 5 Department of Sciences and Biomedical Technologies, University of Milan, Milan, Italy; 6 Milano-Bicocca University, Milan, Italy; 7 Nuclear Medicine Center, San Gerardo, Monza, Italy

**Keywords:** Breast cancer, Primary cell culture, Epithelial-mesenchymal transition (EMT)

## Abstract

**Background:**

Immortalized cancer cell lines are now well-established procedures in biomedicine for a more complete understanding of cellular processes in cancer. However, they are more useful in preparation of fresh tumour tissue, in order to obtain cancer cells with highly preserved individual tumour properties. In the present study we report an analytical investigation on a breast cancer primary cell culture isolated from a surgical specimen obtained from a patient with an infiltrating ductal carcinoma. The objective of the research was to reveal unrecognized aspects of neoplastic cells, typical of the tumour from where the cells were derived, but masked in fixed tissue sections, in order to better predict the aggressive potentiality of the tumour.

**Findings:**

Using a combination of mechanical and enzymatic treatment, the tumour tissue was dissociated immediately after surgical removal. The primary cells were isolated by differential cell centrifugation and grown in selective media. Immunocytochemistry and quantitative RT-PCR analysis were performed to detect the presence of specific biomarkers at protein and transcript level.

The isolated primary breast cancer cells displayed phenotypic behaviour, characteristic of malignant cells and expression of several mesenchymal markers, revealing a strong signature for the epithelial-to-mesenchymal transition associated to a stellate morphology with a number of cellular protrusions and the attitude to overgrow as multilayered overlapping cellular foci.

**Conclusions:**

Our data are a further meaningful indication that primary cell cultures represent a powerful system that could be applied to those cases deserving a deeper investigation at molecular level in order to design individualized anticancer therapies in the future.

## Background

Breast cancer recovery has increased in recent years, thanks to the efforts of research in this field. However, despite the important results obtained, breast cancer remains a complex multifactorial pathology, hard to describe comprehensively and therefore difficult to treat appropriately. Indeed, several subtypes of breast carcinoma have been acknowledged with different clinical outcomes and therapeutic responses. Histological and molecular classification of breast cancer have revealed at least four major subgroups: 1) basal-like: estrogen receptor-negative (ER^-^), progesterone receptor-negative (PR^-^), HER2-negative (HER2^-^); 2) luminal A: estrogen receptor-positive (ER^+^, low grade); 3) luminal B: ER^+^, high grade; 4) HER2-positive (HER2^+^) [1]. All these subgroups generally correspond to different patients’ prognoses and responsiveness to therapy. The HER2^+^ and basal-like phenotypes, for example, exhibit poor prognosis and resistance to therapy, although the HER2^+^ subgroup is usually responsive to targeted therapy with Herceptin. The basal-like tumours show higher initial responses to neoadjuvant chemotherapies [2,3], but show preferential relapse to the brain and to the lung [4,5]. However, these parameters appear still insufficient to give a reliable prevision of the clinical outcome of the individual patient.

A critical point is the prediction of the metastatic potentiality of a tumour. The metastatic project of a carcinoma begins when the neoplastic cells, of epithelial origin, start to detach from the tissue boundaries, lose polarity, the stationary phenotype and acquire motile capability. This process is defined as ‘epithelial-to-mesenchymal transition’ (EMT) and has been described in the past in the morphogenetic events during development. Only more recently, during the 90’s, has EMT been recognized as an instrument for neoplastic cell invasion and metastasis production, including breast cancer cells [6-9]. However, evidence to demonstrate the existence of EMT *in vivo* has been controversial. This is because histological preparation of *ex-vivo* tissues is not sufficiently adequate to reveal the molecular and immunological traits of EMT. To overcome these limitations, cultivation of primary cells from individual breast cancer tissues is a fundamental approach for the *in vitro* study of a wide variety of neoplastic cell properties. In addition, primary cells mimic more closely the *in vivo* behaviour, as compared to the immortalized breast cancer cell lines. On the other hand, the procedure of isolation and the maintaining of primary cells in culture is a good strategy that could be used for those cases deserving a deeper investigation at molecular level to designing anticancer therapies. Here, we report the analytical investigation of a neoplastic cell population isolated from an infiltrating ductal carcinoma (IDC) of the breast. These cells revealed phenotypic traits of a malignant behaviour such as a stellate morphology, cytoplasmic protrusions, multilayered growth in the absence of contact inhibition and expression of several mesenchymal biomarkers, displaying a strong signature for the epithelial-to-mesenchymal transition. The follow up of the patient was not part of this study.

## Materials & methods

### Tissue sample

**Figure 1:**
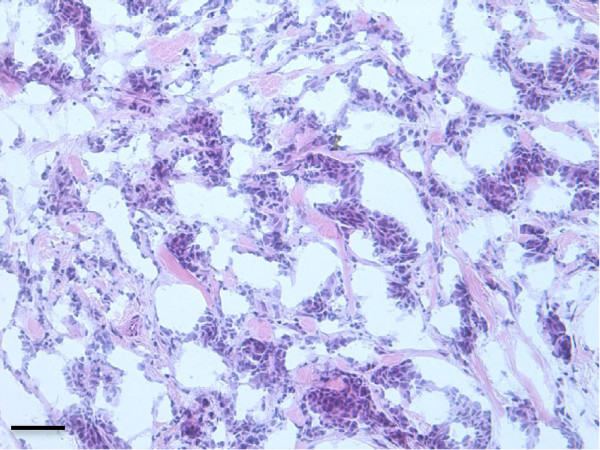
**Representative histological section of the resected IDC tumour sample (H&E, 20x).** Calibration bar: 30 μm.

An extra portion of tissue of an IDC of the breast, unnecessary for histological diagnosis after surgery, was obtained for this study. The patient gave a written informed consent to research partecipation according to the Helsinki Declaration and the study was approved by the Ethical Committee of the San Raffaele G. Giglio Hospital, Cefalù (number of protocol: C.E.2012/16). A representative hematoxylin and eosin (H&E) stained section of the resected tumour is shown in Figure 1.

### Cell culture

The breast tissue was disaggregated mechanically immediately after surgery and then digested overnight at 37°C with a mixture of 1 mg/ml collagenase type I (Invitrogen) and 100 U/ml hyaluronidase (Sigma) in DMEM containing 100 U/ml penicillin and 100 μg/ml streptomycin. DMEM medium and antibiotics were purchased from Invitrogen. Cells were then recovered by differential centrifugation method modified by Speirs et al. [10].

The breast cancer primary cells, named BCpc, were cultured in a selective medium and grown at 37°C under 5% CO_2_-95% air atmosphere in a humidified incubator. The medium composition was the following: DMEM with antibiotics supplemented with 10 mM Hepes (Invitrogen) and 1 mg/ml bovine serum albumin (BSA), 10 ng/ml cholera toxin, 0.5 μg/ml hydrocortisone, 5 μg/ml insulin, 5 ng/ml epidermal growth factor (EGF), 5 μg/ml apo-transferrin (all from Sigma).

**Figure 2:**
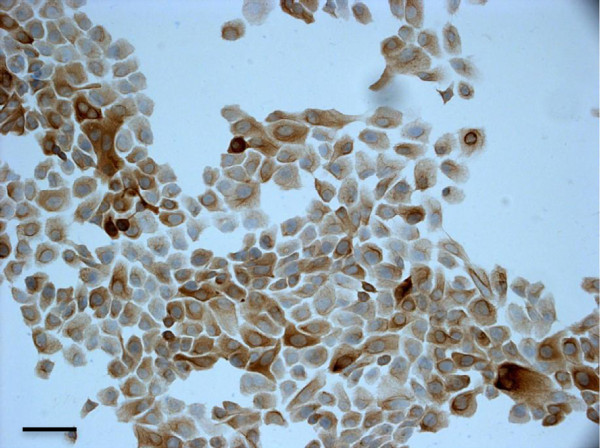
**Light micrographs of the non-tumorigenic MCF-10A epithelial breast cell line immunostained for the epithelial marker panCK (20x).** Calibration bar: 30 μm.

For qRT-PCR experiments, the non-tumorigenic breast epithelial MCF-10A cell line, expressing the epithelial marker pancytokeratin (panCK) was used as control (Figure 2) and cultured according to ATCC instructions.

### Immunocytochemistry (ICC)

To conduct the ICC analysis the breast cancer primary cells (BCpc) were grown on slides until the cells were at sub-confluence and then fixed. Cell fixation was performed using methanol-based buffered preservative solution (ThinPrep PreservCyt Solution) according to the manufacturer’s instructions. ICC analysis was carried out using the Ventana Benchmark automated staining system and the propietary reagents, unless otherwise specified. Endogenous peroxidase activity was blocked utilizing the Ventana inhibitor in the kit. Incubation with primary antibody was carried out at 37°C for 30 minutes followed by washing with PBS (Phosphate-Buffered Saline). The site of the antigen was visualised with RocheVentana’s Ultra View DAB kit. The cells were counterstained with Ventana Haematoxylin and blued with Ventana Blueing Solution. On completion of staining, the cells were dehydrated in alcohol, cleared in Xylene and mounted in synthetic resin. Negative controls, where the primary antibody was excluded, confirmed the specificity of immunostaining. Roche Ventana primary antibodies were used to detect all selected markers, except Ki-67/Mib-1 (DakoCytomation), EGFR (Zymed Laboratories), and CD44 (Neomarkers), according to the manufacturer’s instructions.

### RNA extraction and quantitative RT-PCR analysis

Total RNA was extracted from the BCpc and MCF-10A cells utilizing TRIzol reagent according to the manufacturer’s specifications (Invitrogen). RNA concentration was determined by spectrophotometry. One μg of total RNA was reverse-transcribed into cDNA with SuperScriptII reverse transcriptase (Invitrogen) and 0.5 μg oligo(dT) in a final volume of 20 μL. One μL of cDNA (50 ng RNA equivalent) was analysed by Real-Time PCR (1 cycle 95°C for 20 sec and 40 cycles of 95°C for 3 sec, 60°C for 30 sec) in triplicate using Fast 7500 Real-Time PCR System (Applied Biosystems). Amplification reactions were performed in a 20 μl reaction volume containing 10 pmoles of each primer and the Fast SYBR Green Master Mix according to the manufacturer’s specifications (Applied Biosystems). Reaction specificity was controlled by post-amplification melting curve analysis. The oligonucleotide primers were selected with Primer3 software ( http://fokker.wi.mit.edu/primer3) and tested for their human specificity using NCBI database. Primers sequence (forward and reverse) used are listed in Table 1. Quantitative data, normalized versus rRNA 18S gene, were analysed by average of triplicates Ct (cycle threshold) according to the 2^-ΔΔct^ method using SDS software (Applied Biosystems). The data shown were generated from three independent experiments and the values are expressed relative to mRNA levels in the non-tumorigenic breast epithelial MCF-10A control cells as mean ± SD.

**Table 1:** The sequences of primers selected to perform qRT- PCR analysis

**Gene Name**	**GenBank**	**Forward primer**	**Reverse primer**
*ACTA2* (α-SMA)	NM_001141945.1	5′-gctgttttcccatccattgtg-3′	5′-ttggtgatgatgccatgttct-3′
*CDH1* (E-cadherin)	NM_004360.3	5′-ccaagtgcctgcttttgatga-3′	5′-cccctacccctcaactaac-3′
*CDH2* (N-cadherin)	NM_001792.3	5′-gacaatgcccctcaagtgtt-3′	5′-ccattaagccgagtgatggt-3′
*FN1*(Fibronectin 1)	NM_212482.1	5′-ggaaagcatatgcagccaac-3′	5′-ctacagtattgcgggccaga-3′
*KRT5* (CK5)	NM_000424.3	5′-tttgtctccaccacctcctc-3′	5′-cctgggaaccaaagaatgtg-3′
*KRT6* (CK6)	NM_005554.3	5′-caagctcaccttccaggact-3′	5′-gagtgtgagaggctggagga-3′
*KRT8* (CK8)	NM_002273.3	5′-gggaagctggtgtctgagtc-3′	5′-ctcctgttcccagtgctacc-3′
*KRT18* (CK18)	NM_000224.2	5′-ccagtctgtggagaacgaca-3′	5′-atctgggcttgtaggccttt-3′
*RN18S1*(rRNA18S)	NR_003286.2	5′-aaacggctaccacatccaag-3′	5′-caattacagggcctcgaaag-3′
*SNAI1* (Snail)	NM_005985.3	5′-gcgagctgcaggactctaat-3′	5′-ggacagagtcccagatgagc-3′
*TGFβ1*	NM_000660.4	5′-ccctggacaccaactattgc-3′	5′-aggcagaagttggcatggta-3′
*Twist1*	NM_000474.3	5′-aaactggcctgcaaaaccatag-3′	5′-tgcattttaccatgggtcctg-3′
*VIM* (vimentin)	NM_003380	5′-aacaaccgacactcctacaaga-3′	5′-tggttggatacttgctggaaa-3′
*ZEB1*	NM_001128128.2	5′-gaaaaaccacaaggggatgag-3′	5′-gcttgactttcagccctgtc-3′
*ZEB2*	NM_014795.3	5′-atggggccagaagccacgat-3′	5′-gtcgactgcatgaccatcgc-3′

## Findings

### Isolation and *in vitro* expansion of primary cells from the breast tumour specimen

**Figure 3:**
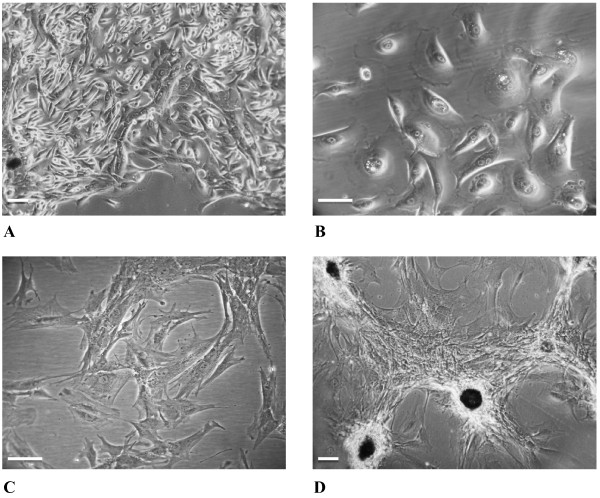
**Phase-contrast micrographs of breast cancer primary cells (BCpc) at different stages of the culture. A**. Initial phase of cell spreading from the tissue after 7 days in culture (10x). **B**. 15-day culture showing the cells fully dissociated (20x). **C**. A detail of the culture at the 4^th^ growth passage showing the stellate morphology and cellular protrusions (spikes) (20x). **D**. The cells forming multilayered foci within 24-48 hrs in culture (10x). Calibration bars: 30 μm.

Primary tumour cells were isolated from breast tissue of a patient with an infiltrating ductal carcinoma (IDC) and with the following histologic features: moderately differentiated, G2, pT2, pN1a, MIB-1 (KI-67) proliferation index 21%, ER^+^/PR^+^, HER2^-^. The tissue was disaggregated mechanically immediately after surgery and the cells were isolated by applying enzymatic digestion with collagenase/hyaluronidase and differential centrifugation method [10]. Following enzymatic treatment and culture in selective media, the breast cancer primary cells (BCpc) started to move away from the tissue boundaries, acquiring a stellate morphology. Figure 3 shows a panel of phase-contrast micrographs of cell culture fields at different growth stages: the initial phase of cell spreading from the tissue after 7 days in culture (Figure 3A); a field of fully dissociated cells after 15 days (Figure 3B); a detail of cells at the 4^th^ passage manifesting the stellate morphology and a number of cellular protrusions, or spikes (Figure 3C); within 24-48 hrs in the culture, the cells at the 4^th^ growth passage were fully widespread, adjoined to each other and overgrown in scattered dense foci of multilayered overlapping cells (Figure 3D).

### Immunological characterization

**Figure 4:**
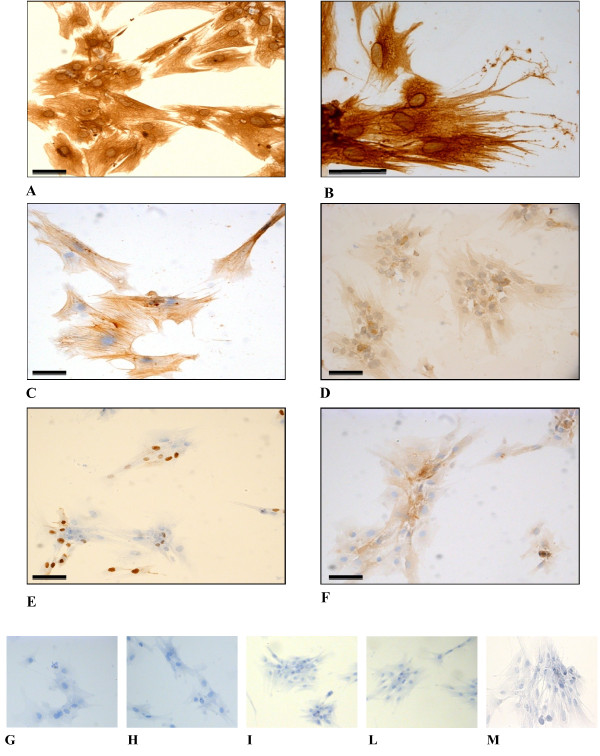
**Light micrographs of BCpc immunostained for A. vimentin (25x) and B. (50x); C. SMA (25x); D. EGFR (25x); E. Ki-67 (25x); F. CD44 (25x).** Calibration bars: 30 μm. Cropped areas of representative fields of negative controls for: **G**. vimentin, **H**. SMA, **I**. EGFR, **L**. Ki-67, **M**. CD44.

**Figure 5:**
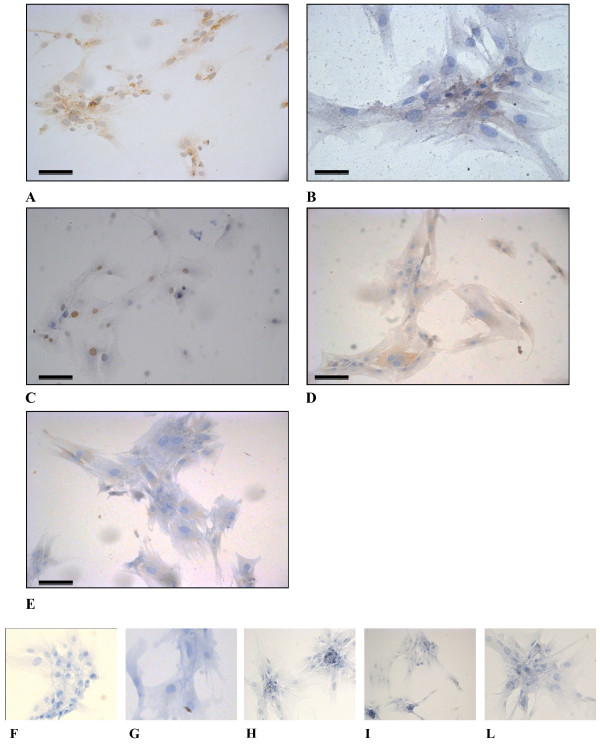
**Light micrographs of BCpc immunostained for A. panCK (25x); B. CK8/18 (50x); C. p53 (25x); D. p63 (25x); E. c-kit (25x). Calibration bars: 30 μm.** Cropped areas of representative fields of negative controls for: **F**. panCK, **G**. CK8/18, **H**. p53, **I**. p63, **L**. c-kit.

**Figure 6:**
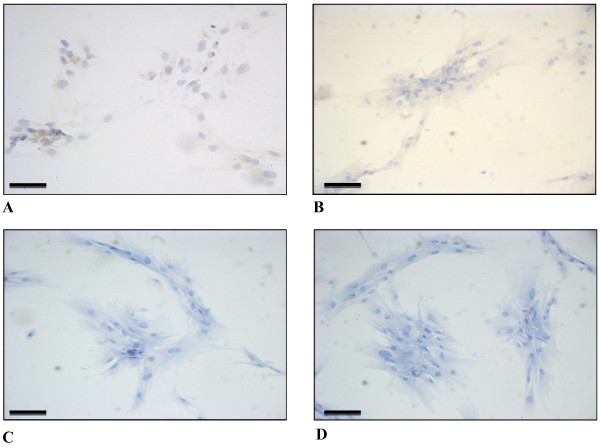
**Light micrographs of BCpc immunostained for A. CK5/6 (25x); B. HER2 (25x); C. ER (25x); D. PR (25x).** Calibration bars: 30 μm.

Cells were cultured for 4 passages and then assayed to test the expression of known breast cancer biomarkers. The ICC analysis revealed a complex immunological pattern that did not fall into the major subtypes. A panel of micrographs of ICC images of BCpc grown on slides and incubated with the proper antibody is reported in Figure 4. Based on immuno-staining intensity, the reaction was classified as positive for vimentin, alpha-smooth muscle actin (α-SMA), EGFR, Ki-67, and for stem cell marker CD44 (Figure 4A-F); weakly positive for pancytokeratin (panCK), luminal CK8/18, and for basal-like markers p53, p63 and c-kit (Figure 5A-E); negative for basal CK5/6, HER2, ER and PR (Figure 6A-D). While the cytokeratin reaction was rather faint, the SMA and in particular, vimentin staining was very intense and its localization made it possible to appreciate the long cell projections, frequently making contacts between one cell and the neighbouring one. In addition, from the tip of some spikes it was also possible to observe the release of vesicles, putative exosomes (Figure 4B). These data were indicative of a strong signature for the epithelial-to-mesenchymal transition.

### Transcriptional analysis

**Figure 7:**
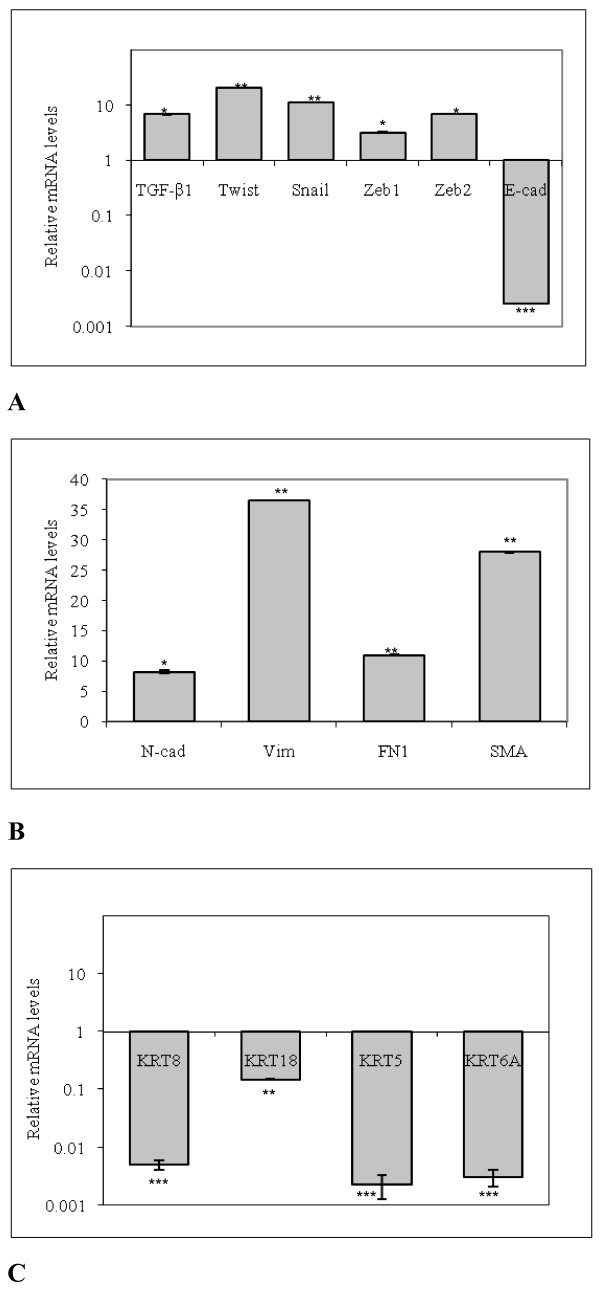
**BCpc express multiple genes associated with an epithelial mesenchymal transition as determined by qRT-PCR.** The gene expression data are relative to the mRNA levels in MCF-10A control cells and are mean ± SD of three independent experiments (***p < 0.001, **p < 0.01, *p < 0.05, all versus control cells).

To test if the observed phenotypic and immunological traits of the BCpc could be reconducted to the epithelial-mesenchymal transition, quantitative RT-PCR (qRT-PCR) analyses were performed in parallel with non-tumorigenic mammary epithelial MCF-10A cells, used as a normal control for *in vitro* studies of breast cancer [11,12]. The genes selected were some of the most representative targets of the TGF-β signal transduction pathway, as well as the cytoskeleton and adhesion proteins, the downstream targets of the pathway [13,14]. Relative to the non-tumorigenicMCF-10A cells, in the BCpc we observed a significantly increased expression of transcriptional levels of TGF-β1 gene (~6.8-fold), a major inducer of the EMT (Figure 7A). Moreover qRT-PCR analysis revealed an increase of mRNA levels of some transcriptional factors, which are the master regulators of EMT. In particular, Twist was 20.9-fold higher, Snail 11.4-fold, and Zeb1 and Zeb2 3.2-fold and 6.8-fold, respectively, suggesting an induction of these factors by TGF-β signalling pathway. Since all these genes contribute to transcriptional repression of the E-cadherin gene (CDH1), as expected, BCpc showed a downregulation of this gene (Figure 7A). In addition, the BCpc expressed high levels of the mRNAs which encode mesenchymal markers, the final targets activated by the TGF-β pathway, specifically N-cadherin (CDH2, ~8.2-fold), Vimentin (VIM, ~36.5-fold), Fibronectin (FN1, ~11-fold), and α-SMA (ACTA2, ~28-fold), which contribute to the establishment of the motile and invasive cell phenotype (Figure 7B). As the induction of mesenchymal factors by the TGF-β is associated with the downregulation, beyond the E-cadherin, of various epithelial factors including several keratins [12], we also assayed the expression of Cytokeratin 8 (KRT8), 18 (KRT18), 5 (KRT5) and 6 (KRT6A) genes in the BCpc. The qRT-PCR results showed a significant downregulation of transcriptional levels of all these genes in our cellular system (Figure 7C).

In general, these data indicate that the BCpc expressed multiple genes associated with an epithelial-mesenchymal transition.

## Discussion

The application of cell culture to the clinical research has received great impulse in recent years with the acknowledgement that cells derived from different normal and tumorous tissues are able to maintain most of their proper phenotypic traits, as a memory of their origin, especially the primary cells cultures. In addition, primary cultures represent a powerful system to study cell behaviour and cellular pathways. It is well documented that epithelial cells derived from normal tissues maintain in culture a polarized stationary phenotype and show contact inhibition during proliferation. On the contrary, cancer cells usually display non-polarized cell morphology, reduction or absence of intercellular contacts and a motile attitude which is typical of mesenchymal cells.

The aim of this study was to identify phenotypic traits and molecular markers in primary cell culture derived from a patient with IDC of the breast, useful to evaluate risk of tumour progression *in vivo*. After tumour tissue dissociation and cell spreading, the totality of viable cells displayed the characteristic features of malignant cells: that is a markedly irregular morphology, often of a stellate aspect with extensive cellular protrusions. Immunocytochemistry assays revealed a complex immunological pattern. The majority of cells were negative for HER2 and for estrogen and progesterone receptors, as expected for cells undergoing EMT [15]. Moreover, the cells were weakly positive for the basal-like biomarkers p53, p63 and c-kit, and generally positive for EGFR, Ki-67 and for stem cell marker CD44.

Concerning the cytoskeleton proteins, the cells were weakly positive for pancytokeratin (panCK), that represents a general marker for cells of epithelial origin, also weakly positive for the luminal cytokeratins (CK8/18) and negative for the basal ones (CK5/6). On the contrary, all cells were strongly positive for vimentin, the mesenchymal cytoskeletal protein, and for the alpha-smooth muscle actin (α-SMA). These results well support the occurrence of the phenotypical EMT process in the cells of our study. To reinforce this hypothesis, a further set of investigations were conducted in order to evaluate the transcription levels of the already detected biomarkers and to expand the panel of candidate genes, by the use of qRT-PCR. Literature data report that different pathways are expected to control the EMT during tumour growth, in the first place the TGF-β-driven cascade [13,14]. It is now generally accepted that elevated TGF-β level is suppressive during the early phase of tumour outgrowth, while at later stages it amplifies malignant conversion and tumour progression. Consistent with the response to TGF-β, the majority of breast tumours, including their metastases, are positive for nuclear factors activated by TGF-β signalling pathway [16-19]. In addition, TGF-β is reported to promote resistance to apoptosis, as well as to induce and initiate the EMT cascade [20]. Interestingly, in our system we found a 6.8-fold increase of TGF-β1 mRNA level with respect to the non-tumorigenic control cells. Concurrently, the transcription factors downstream of Smad, i.e. Twist, Snail, Zeb1 and Zeb2 were increased several fold in comparison to the control cells, indicating an active transcription of factors involved in the TGF-β signalling pathway. Significantly, all these genes concur to transcriptional repression of E-cadherin, whose down-regulation progressively causes cell detachment and the loss of the apico-basal polarity [13,14].

What appeared confirmatory of the TGF-β pathway in our study was the down-regulation of the E-cadherin transcription gene coupled with a significant increment of the N-cadherin expression.

It is known that in breast cancer progression the loss of E-cadherin–based cell adhesion is an important factor in tumour invasiveness and that an over-expression of another adhesion molecule, the N-cadherin, is associated with an increased invasive potential of tumour cells [21-25]. Moreover, N-cadherin contributes to induce a mesenchymal-scattered phenotype associated with reduced E- and P-cadherin levels in squamous cell carcinoma and to promote breast cancer cell migration, invasion and metastasis [26-30]. In our case, the hypothesis of the EMT occurrence was further validated by the observed transition in the cytoskeleton assembly. Indeed, in addition to cytokeratin down-regulation a net increment of vimentin transcription appeared, together with the ACTA2 (α-SMA) and fibronectin. A remarkable observation was the radiating immune-reaction of vimentin from the perinuclear area to the tip of long cytoplasmic protrusion, correlating with the dramatic cell changes from epithelial to mesenchymal shapes and from stationary to motile phenotype, as also decribed by other Authors [6]. Besides the classical role of cytoskeletal protein, recent studies have attributed several key functions to the vimentin, which now appears to be an organizer of a number of critical functions involved in attachment, migration, and ramification of cell signalling into several aspects of cell physiology and pathology [31-33]. In addition, vimentin is also considered as a novel potential anti-cancer therapeutic target [34].

Our results and literature data, support the idea that the full manifestation of the invasive properties of tumour cells is accomplished when cells are fully detached from the neighbouring ones, losing cell-cell and cell-matrix interactions. These described changes are indicative of an epithelial-to-mesenchymal transition which represents a terminal point of a cascade of events, at transcriptional and translational level, inducing severe derangement of the original tissue architecture and a reprogramming of the neoplastic phenotypes. The EMT phenomenon is difficult to predict in its dynamic evolution inside tissue sections, while the application of primary culture is a way to unmasking all the process.

## Conclusion

As reported, we suggest that tissue dissociation and culturing of primary cells after surgical removal of the tumour offer great possibilities to investigate malignant potentiality of tumour cells in addition to those that the tissue section may provide. We trust that this study may offer a basis for further characterization of the molecular mechanisms of breast cancer progression and for designing individualized anticancer therapies in the future.

## Abbreviations

IDC: Infiltrating ductal carcinoma; EMT: Epithelial-mesenchymal transition; ICC: Immunocytochemistry; BCpc: Breast cancer primary cells; qRT-PCR: Quantitative reverse transcription polymerase chain reaction; ER: Estrogen receptor; PR: Progesterone Receptor.

## Competing interests

The authors declare that they have no competing interests.

## Authors' contributions

All authors participated to the conception, design, interpretation and elaboration of the findings of the study. All authors read and approved the final manuscript.
